# Characterization of Autoantibodies against the E1**α** Subunit of Branched-Chain 2-Oxoacid Dehydrogenase in Patients with Primary Biliary Cirrhosis

**DOI:** 10.1155/2012/369740

**Published:** 2012-06-20

**Authors:** Tsutomu Mori, Hiromasa Ohira, Masahito Kuroda, Masaki Kato, Yoshiki Yamaguchi, Hideo Kochi

**Affiliations:** ^1^Department of Human Lifesciences, Fukushima Medical University School of Nursing, 1 Hikarigaoka, Fukushima 960-1295, Japan; ^2^Department of Internal Medicine II, Fukushima Medical University School of Medicine, 1 Hikarigaoka, Fukushima 960-1295, Japan; ^3^Structural Glycobiology Team, Systems Glycobiology Research Group, Chemical Biology Department, RIKEN Advanced Science Institute, 2-1 Hirosawa, Wako, Saitama 351-0198, Japan; ^4^Department of Biochemistry, Fukushima Medical University School of Medicine, 1 Hikarigaoka, Fukushima 960-1295, Japan

## Abstract

Primary biliary cirrhosis (PBC) is characterized by antimitochondrial antibodies (AMAs) that react with the lipoyl-containing E2 subunits of 2-oxoacid dehydrogenase complexes such as BCOADC and PDC. The lipoyl domains of E2 contain the major epitopes essential for immunopathology. However, the non-lipoyl-containing E1 subunits are also frequently targeted. Since anti-E1 antibodies always appear in combination with anti-E2 antibodies, the mechanisms underlying the autoimmunity against E1 may be linked to, but distinct from, those against E2. Here, we demonstrate that intermolecular and intramolecular determinant spreading underlies the autoimmunity against E1. We performed characterizations and epitope mapping for anti-BCOADC-E1*α* antibodies from both the intermolecular and intramolecular points of view. The antibody reactivities form a cluster against the BCOADC complex that is distinct from that against the PDC complex, and the anti-BCOADC-E1*α* antibodies arise as part of the cluster against the BCOADC complex. Multiple epitopes are present on the surface of the BCOADC-E1*α* molecule, and the major epitope overlaps with the active center. Sera with anti-BCOADC-E1*α* antibodies strongly inhibited the enzyme activity. These findings suggest that the E1*α* subunit as part of the native BCOADC complex is an immunogen, and that determinant spreading is involved in the pathogenesis of AMA production.

## 1. Introduction

Primary biliary cirrhosis (PBC) is an autoimmune disease, and high-titer antimitochondrial autoantibodies (AMAs) are characteristically present in sera from almost all PBC patients [1, 2]. The major autoantigens recognized by AMA are the lipoyl-containing E2 subunits of 2-oxoacid dehydrogenase complexes [3], such as the branched-chain 2-oxoacid dehydrogenase complex (BCOADC) and pyruvate dehydrogenase complex (PDC). The multimeric E2 subunits form the structural core of each complex, around which multiple E1 and E3 subunits assemble to form huge macromolecular complexes. In PDC, there is another subunit, termed E3BP, which is involved in the association of E3 with the E2 core. The lipoyl domain of the E2 polypeptide contains the major epitopes recognized by both AMA and T cells [4–7], and the significance of these epitopes in eliciting autoimmunity has been firmly established [8]. Curiously, however, the non-lipoyl-containing E1 components (and other subunits) are also recognized by AMA [9, 10]. More recently, the frequencies of antibodies against the *α* and *β* subunits of PDC-E1 have been reported to be strikingly high [11], indicating the significance of anti-E1 autoimmunity in the pathogenesis of AMA. Furthermore, anti-E1 antibodies are exclusively found in sera from patients with anti-E2 antibodies [9–12], suggesting antibody diversification from E2 to E1. Therefore, the mechanisms underlying anti-E1 autoimmunity may be closely linked to, but clearly distinct from, those of anti-E2 autoimmunity. Although such features may reflect the pathogenesis of AMA, the potential significance of anti-E1 autoimmunity has been largely overlooked [11, 13].

Determinant spreading refers to the development of immune responses against endogenous epitopes as a result of tissue damage [14] and plays an active role in ongoing disease pathology [15]. Therefore, it is necessary to clarify whether a determinant spreading cascade also underlies the pathogenesis of PBC. In this regard, the reactivity of AMA against the non-lipoyl-containing E1 components is suggestive of determinant spreading. However, since very little attention has been paid to the immunogenic roles of E1 proteins [11, 13], the involvement of a spreading cascade in PBC has not been argued extensively. To address this issue, analyses of antibody reactivity against E1 should be carried out in comparison with those against other antigens. In addition, detailed epitope mapping of the E1 antigens is necessary. We previously characterized the autoreactivity against non-lipoyl-containing subunits by focusing on the E1*α* molecules, which are the most frequent targets of AMA in this category [10, 16]. In the present study, we analyzed the relationships between the antibody reactivity against BCOADC-E1*α* and those against other subunits of BCOADC and PDC. In addition, we carried out antibody epitope mapping of BCOADC-E1*α* using a multipin ELISA [17], which has been applied to systematically search for epitopes on various antigens [18–20]. The solvent accessibilities of the epitopes were analyzed based on the reported crystal structure of BCOADC-E1 [21]. Our findings revealed that anti-BCOADC-E1*α* antibodies appeared in association with antibodies against other subunits of BCOADC but not of PDC. In addition, the major epitope overlapped with the active center, and multiple B-cell autoepitopes were mapped on the surface of the BCOADC-E1*α* molecule. These results suggested the involvement of both intermolecular and intramolecular determinant spreading. Thus, a spreading cascade may underlie the pathogenesis of AMA, similar to the case for other autoimmune diseases.

## 2. Materials and Methods

### 2.1. Sera

PBC sera were collected from 30 patients with a well-established diagnosis of PBC. During testing with human liver BCOADC-E1*α*, 16 of the 30 PBC sera were positive by ELISA and western blot analysis as previously described [10]. We used these 16 sera for epitope mapping of BCOADC-E1*α*, all of which were at the time point of first diagnosis. Sera from 10 healthy volunteers were employed as controls. All the subjects were from clinics at Fukushima Medical University (Fukushima, Japan), and provided written informed consent. The study protocol was approved by the Ethical Committee of Fukushima Medical University and conformed to the Ethical Guidelines of the 1975 Declaration of Helsinki.

### 2.2. Materials

Blocks with pins were purchased as part of a Mimotope epitope scanning kit (Chiron, Clayton, Australia). Fmoc-protected amino acids and dimethylformamide were obtained from Wako Pure Chemical Industries Ltd. (Osaka, Japan). Affinity-purified goat anti-human IgG conjugated with horseradish peroxidase was purchased from Bio-Rad Laboratories (Richmond, CA). A 3′,3′,5′,5′-tetramethylbenzidine peroxidase substrate kit for ELISA was obtained from Sumitomo Bakelite (Tokyo, Japan). BlockAce was purchased from Dainihon Seiyaku (Osaka, Japan). All other chemicals used were of the highest grade available from commercial sources.

### 2.3. Purification of the Subunits of Human Liver BCOADC

Human liver BCOADC was purified using phenyl-Sepharose chromatography as described previously [10]. The obtained E1, E2, and E3 components of the complex were further subjected to preparative SDS-PAGE. Proteins were visualized by zinc staining [22], and the individual areas corresponding to BCOADC-E1*α* (46 kD), BCOADC-E1*β* (36 kD), BCOADC-E2 (52 kD), and BCOADC-E3 (55 kD) were excised and recovered by electroelution [23] using an AE-6580 electroelution apparatus (ATTO, Tokyo, Japan). The purity of each polypeptide was verified by SDS-PAGE followed by silver staining. The subunits of PDC were purified as described previously [10].

### 2.4. Enzyme Inhibition Assay

The BCOADC activity was reconstituted in vitro by mixing the purified E1, E2, and E3 components as described previously [24]. Before assay, 1.25 *μ*g of E1 was incubated for 3 min on ice with sera at a 200-fold dilution.

### 2.5. ELISA

The reactivities of the sera against purified BCOADC-E1*α* were determined by ELISA in MS-3696F 96-well immunoplates (Sumitomo Bakelite). Briefly, 50 ng of a purified antigen was added to each well and blocked with 10% Block Ace in PBS overnight. Sera diluted 1 : 100 to 1 : 1000 with PBST containing 10% Block Ace were added to the wells and incubated at room temperature for 1 h. After four washes with PBST, horseradish peroxidase-conjugated goat anti-human IgG diluted 1 : 1000 in PBST was added to each well and incubated for 1 h. Next, the wells were washed four times with PBST, and color development was initiated by the addition of 100 *μ*L of 3′,3′,5′,5′-tetramethylbenzidine for 5 min and terminated by the addition of 100 *μ*L of 2 N H_2_SO_4_. For statistical analyses, SPSS software (SPSS, Chicago, IL, USA) was used. Cluster analysis was carried out by the nearest neighbor method.

### 2.6. Synthesis of Peptides

Overlapping peptides were synthesized according to the BCOADC-E1*α* sequence (GenBank, NP_000700). In the following procedure, the Ser^46^ residue just after the mitochondria-targeting sequence was designated amino acid number 1. Peptides were synthesized on a 96-pin block with the aid of the Mimotope software as previously described [16, 17]. Peptide synthesis was verified by the reactivities of control antibodies supplied by the manufacturer against positive and negative control peptides (PLRQ and GLAQ, resp.), both of which were synthesized on every pin block.

### 2.7. Multipin ELISA

The reactivities of the sera against the synthesized peptides were estimated by pin ELISA as previously described [16]. The bound antibodies were estimated using a model 3550 microplate reader (Bio-Rad Laboratories). The results of the multipin ELISA were subjected to statistical analyses [25]. The absorbance value for each peptide was transformed into a standard score (*Z*-score) that represented the magnitude of deviation from the mean absorbance value. A *Z*-score of >2.0 was considered significant.

### 2.8. Mapping of the Epitopes on the Crystal Structure of BCOADC-E1*α*

Crystallographic data for BCOADC-E1 (PDB, 2bev [21]) were utilized for analyses. The solvent-accessible surface areas of the BCOADC-E1*α* subunit were calculated using the DSSP program (http://swift.cmbi.ru.nl/gv/dssp/) [26]. Visualization of epitopes on the crystal structure of BCOADC-E1 was conducted with the PyMOL software (http://www.pymol.sourceforge.net/; DeLano Scientific, South San Francisco, CA, USA).

### 2.9. Homology Analysis

Sequence alignment of amino acids between human BCOADC-E1*α* and human PDC-E1*α* was conducted using the BLAST2 Program (http://www.ncbi.nlm.nih.gov/blast/bl2seq/wblast2.cgi/) [27]. Homology analyses of amino acids among BCOADC-E1*α* subunits from various species were performed by aligning the protein sequences using the ClustalW2 program (http://www.ebi.ac.uk/Tools/clustalw2/) [28]. Thereafter, the homology index of each residue was calculated by the ConSurf program (http://consurf.tau.ac.il/) [29] on the basis of X-ray diffraction data (PDB, 2bev [21]). The accession numbers for the analyzed proteins are listed in the Supplemental Methods.

## 3. Results

### 3.1. The Antibody Reactivities Form a Cluster against BCOADC That Is Distinct from That against PDC

**Figure 1:**
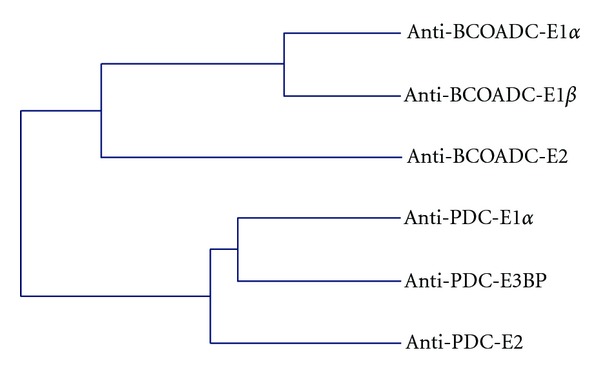
Interrelationships among antibodies against each subunit. Dendrogram was obtained by cluster analysis of the relationships among the antibody reactivities in PBC sera against the individual antigenic subunits of BCOADC and PDC. The cluster analysis was conducted using the nearest neighbor method based on the Spearman correlation coefficients in Table 2 as the similarity indices.

Initially, the antibody profile against BCOADC-E1*α* in sera from patients with PBC was compared with that against BCOADC-E2. As shown in Table 1, anti-BCOADC-E1*α* antibodies were always found with anti-BCOADC-E2 antibodies. Based on Fisher's exact test, the appearance of anti-BCOADC-E1*α* antibodies was significantly linked to that of anti-BCOADC-E2 antibodies (*n* = 30, *P* = 0.037). Such a relationship was also found between the appearances of anti-PDC-E1*α* and anti-PDC-E2 antibodies, and this was also statistically significant (Supplemental Table S1, available online at doi:10.1155/2012/369740  *P* = 0.004). Hence, anti-E1*α* antibodies for each type of complex were always found in combination with antibodies against the respective E2 subunit, which was consistent with previous observations including ours [9–12]. Considering that multiple E1 and other components constitute the complex assembly around the E2 core in vivo, there may be additional relationships between the antibody titers against the E1*α*, E2, and other antigenic subunits. Therefore, we further characterized the relationships among the antibody titers (OD values by ELISA) against each antigenic subunit of both complexes (Table 2). The antibody titer against BCOADC-E1*α* was strongly correlated with that against BCOADC-E1*β* (*n* = 30, *r* = 0.786, *P* = 0.018), and weakly but significantly correlated with that against BCOADC-E2 (*r* = 0.336, *P* = 0.043). However, the antibody titer against BCOADC-E1*α* did not show any significant correlations with those against PDC-E1*α* (*r* = 0.193, *P* = 0.168), PDC-E2 (*r* = 0.073, *P* = 0.358), or PDC-E3BP (*r* = 0.044, *P* = 0.413). Therefore, the antibody titer against BCOADC-E1*α* was correlated with those against the other subunits of the BCOADC complex but not with those against the subunits of the PDC complex. On the other hand, the antibody titer against PDC-E1*α* was weakly but significantly correlated with that against PDC-E2 (*r* = 0.339, *P* = 0.042), and moderately but very significantly correlated with that against PDC-E3BP (*r* = 0.655, *P* = 0.000). However, the antibody titer against PDC-E1*α* did not show any significant correlations with those against the individual subunits of BCOADC. Thus, the antibody titer against each E1*α* subunit was correlated with those against the other antigenic subunits of the complex it belonged to. Cluster analysis (Figure 1) further revealed that there were two identifiable epitope clusters: one toward the BCOADC complex and the other toward the PDC complex. The distance in the relationship between the two antibody clusters was further than the distances between the antibody reactivities toward the individual subunits in each complex. These observations suggest that anti-E1*α* antibodies arise in association with the intermolecular diversification of epitopes among the subunits within each individual complex.

### 3.2. Multipin ELISA Identifies Multiple Epitopes on BCOADC-E1*α*

**Figure 2:**
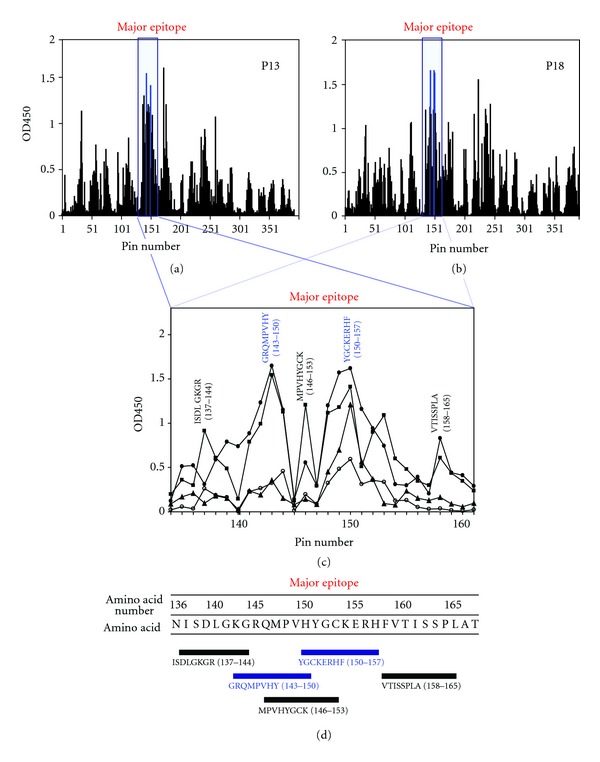
Reactivities of PBC sera against synthesized overlapping peptides of BCOADC-E1*α*. (a) and (b) Pin ELISA profiles of PBC sera. The reactivities of two high-titer PBC sera (P13 and P18) against the peptides were analyzed by pin ELISA. The location of the major epitope (region 6) is indicated by the blue squares. The two peptides showing the highest OD450 values are also highlighted in blue. (c) Clustering of small epitopes in the major determinant region. In the major determinant region (blue square), which corresponds to the blue squares in (a) and (b), multiple small epitopes are present as shown by the multiple peaks in the ELISA profiles. The reactivities of PBC sera against the peptides are shown (○, P07; ■, P13; •, P18; △, P20). The two octapeptides showing the highest values in (a) and (b) are highlighted in blue. (d) Overlapping epitopes are clustered in the major determinant region. The sequences of the octapeptides that exhibited peaks in (c) are indicated. The two octapeptides with the highest values in (c) are highlighted in blue.

In the next step, we carried out a multipin ELISA to better define the antigenic characteristics of BCOADC-E1*α*. The anti-BCOADC-E1*α*-positive sera were selected for epitope mapping, and representative profiles are shown in Figures 2(a) and 2(b). The sera from two symptomatic patients with the strongest titers (P13 and P18) showed similar reaction patterns. The highest reactivity was observed against the region containing amino acids 134–168 with *Z*-scores of more than 3.0 (Supplemental Figure S1), and all the PBC sera with anti-BCOADC-E1*α* antibodies reacted with this region (Table 3). Therefore, this region was regarded as the major determinant on BCOADC-E1*α*. The significance of this region was also supported by the characteristic antibody-binding profiles as follows. As shown in Figure 2(c), multiple small epitopes were clustered in the major epitope region. A similar situation was commonly observed among the anti-BCOADC-E1*α* antibody-positive sera including those from asymptomatic patients. The highly reactive octapeptides with peak OD values in the major determinant region were ISDLGKGR (aa 137–144), GRQMPVHY (aa 143–150), MPVHYGCK (aa 146–153), YGCKERHF (aa 150–157), and VTISSPLA (aa 158–165) (Figure 2(d)). These profiles revealed that the major determinant comprised overlapping epitopes, thus forming “nested epitopes.” This feature suggests that active immune responses target this region.

In addition to the major determinant, multiple regions (designated epitopes 1 to 12) were identified (Table 3). Among these, the major epitope (epitope 6) was invariably recognized, while the reactivities against the remaining epitopes were variable among the sera. The P13 serum, which showed the highest titer against BCOADC-E1*α*, reacted with most of the epitopes. The octapeptides exhibiting the peak OD values within each region, which represent the core sequences, are shown in Supplemental Table S2.  Supplemental Table S2 also indicates the presence of additional epitope clusters in regions 7 and 9. In summary, the above data suggest the occurrence of intramolecular diversification of epitopes on the BCOADC-E1*α* molecule.

### 3.3. Characterization of the Epitopes on BCOADC-E1*α* with Crystallographic Information

**Figure 3:**
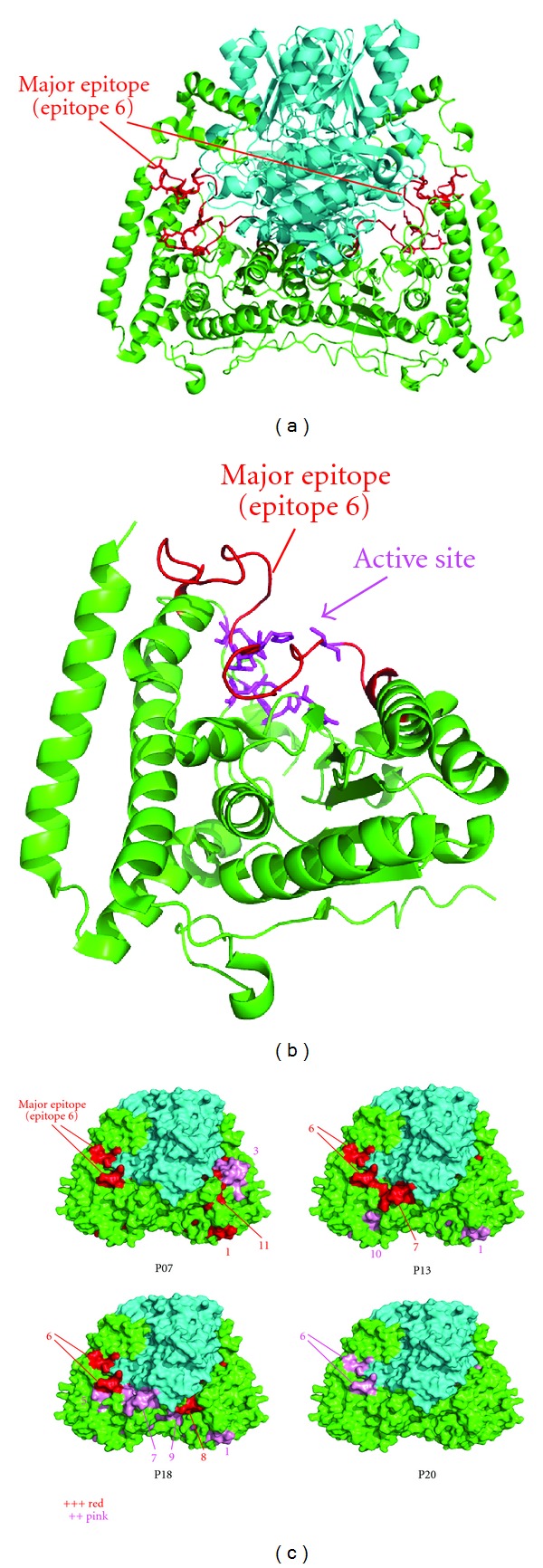
Three-dimensional mapping of the epitopes of BCOADC-E1*α*. (a) Structural visualization of the major determinant (epitope 6) of BCOADC-E1*α*. A three-dimensional structure of the E1 *α*_2_ *β*_2_ heterotetramer [21] is shown with the E1*α* subunit in green and the E1*β* subunit in cyan. The major determinant of E1*α* is shown in red, with stick representations of the side chains of amino acids 137–142 and 153–156. (b) The major determinant overlaps with the active center pocket of E1*α* [21]. The major determinant (epitope 6, aa 134–168) is shown in red, while the residues in the active site are shown in magenta with stick representation. (c) Surface representations of the epitopes of E1*α*. The epitopes on the molecular surface of E1*α* are drawn with the epitope numbers. The strengths of the antibody reactivities are indicated in red and pink, which correspond to +++ and ++ in Table 3, respectively.

Subsequently, the epitopes determined by ELISA were analyzed using crystallographic data for BCOADC-E1 [21]. Using the DSSP program [26], the solvent accessibilities of each residue were calculated. The major determinant (aa 134–168) was shown to contain two solvent-accessible surface areas on the BCOADC-E1 *α*_2_ *β*_2_ tetramer (Supplemental Figure S2). These two areas (aa 137–142 and 153–156) were included within two of the defined epitopes (aa 137–144 and 150–157) that constitute the major determinant region (Figure 2(d)). These observations support the validity of pin ELISA for analyzing epitopes, and suggest that these areas are the primary recognition sites.  Figure 3(a) depicts a three-dimensional map of the major determinant on BCOADC-E1*α*. The major determinant overlapped with the active center (Figure 3(b)). These observations imply that the major determinant may be structurally important for the enzymatic function. Indeed, the sera with anti-BCOADC-E1*α* antibodies strongly inhibited the enzyme activity in an in vitro reconstitution assay (Supplemental Figure S3). Next, the dominant and subdominant epitopes were structurally mapped (Supplemental Figure S4). Although the locations of the epitopes varied among the patients, the epitopes formed weak clusters in each case. Using surface representation (Figure 3(c)), the epitopes were found to form clusters on the surface of the molecule, suggesting that the antibodies recognize consecutive surface areas of BCOADC-E1*α*. Taken together, these results suggest that the native BCOADC-E1*α* itself is an immunogen.

### 3.4. Structural Relationships between BCOADC-E1*α* and Its Homologous Proteins

**Figure 4:**
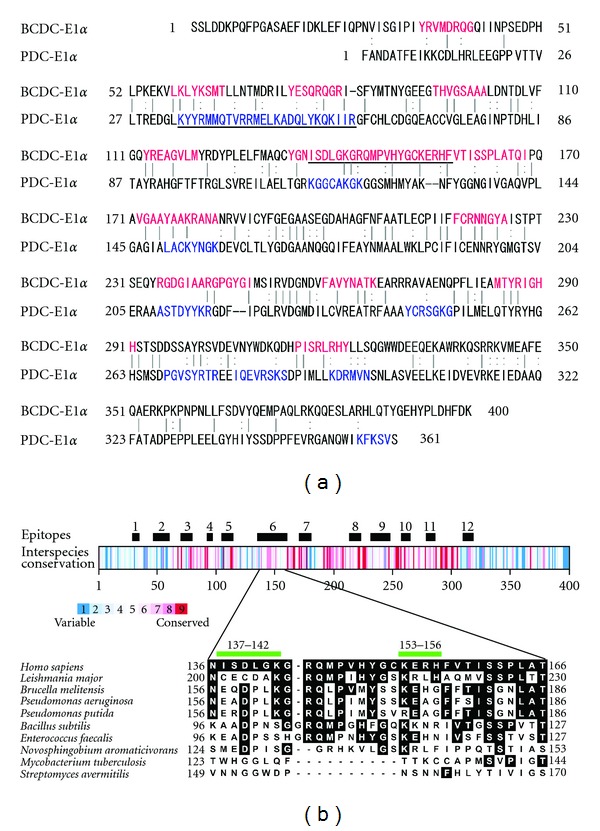
Comparative analyses between the epitopes and the conserved structures of BCOADC-E1*α*. (a) Comparison of the epitopes between BCOADC-E1*α* (BCDC-E1*α*) and PDC-E1*α*. The amino acid sequences of BCOADC-E1*α* and PDC-E1*α* were aligned using the BLAST2 Program [27]. Vertical lines represent identical residues, while dots denote similar residues. The epitopes of BCOADC-E1*α* and PDC-E1*α* determined by the multipin ELISA are indicated in pink and blue, respectively. The major determinant region is underlined for each antigen. (b) Comparison of the epitopes and the interspecies conservation of BCOADC-E1*α*. (a) Upper: interspecies conservation was calculated by the ConSurf program [29] with the aid of the ClustalW2 program [28]. The epitope regions are also indicated. The conservation indices for the individual amino acid residues are drawn in graded colors (blue to red) as indicated. (b) Lower: sequence alignments of the major determinant region of human BCOADC-E1*α* and homologous proteins from various infectious microorganisms. Inverted letters represent residues that are identical to the human protein. The two regions (aa 137–142 and 153–156) that exhibited high solvent accessibilities (Supplementary Figure S2) are also indicated.

With respect to the mechanisms underlying the antibody diversification, one possibility is that it results from antibody cross-reactivity against homologous proteins. Initially, human PDC-E1*α* should be considered to be responsible for initiating such cross-reactivity as a candidate homolog of BCOADC-E1*α*. Both of these molecules are thiamine pyrophosphate-dependent enzymes involved in the first reaction step for oxidative decarboxylation of 2-oxoacids. They share conserved motifs, namely a tetramer interface, thiamine pyrophosphate-binding site, heterodimer interface and phosphorylation loop region [21]. Therefore, antibody cross-reactivity may occur between these two E1*α* proteins. However, no significant relationship between the antibody titers against BCOADC-E1*α* and PDC-E1*α* was found (Table 2). In addition, the appearances of anti-BCOADC-E1*α* and anti-PDC-E1*α* antibodies were statistically independent of one another (Table 4, *P* = 0.466 by Fisher's exact test).  Figure 4(a) shows an alignment of the sequences of human BCOADC-E1*α* (BCDC-E1*α*) and PDC-E1*α* by the BLAST2 program [27], in which the epitope regions are colored in red and blue [16], respectively. A comparison of the epitopes between the two proteins indicated that they were independent of their counterparts. These data compare well with our previous findings that autoantibodies against BCOADC-E1*α* and PDC-E1*α* did not cross-react with each other's antigen [10]. Thus, these antibodies arose in an independent manner.

Finally, the amino acid sequence of human BCOADC-E1*α* was compared with those of its homologs from other species using the ConSurf program [29]. We found that the epitope regions were independent of the conserved segments (Figure 4(b), upper). In addition, the two core sequences of the major determinant (aa 137–142 and 153–156) were specific to the human protein. In this regard, and in contrast to the highly conserved lipoyl-containing E2 subunit, the amino acid sequence of the non-lipoyl-containing E1*α* subunit was less conserved among species (Figure 4(b), lower). These observations support the idea that the immune system in patients targets the self BCOADC-E1*α* antigen.

## 4. Discussion

Our study has demonstrated that both intermolecular and intramolecular diversifications of autoimmunity are associated with the emergence of anti-BCOADC-E1*α* antibodies. Although it is difficult to chase the autoimmune profiles over time from the beginning of the pathogenesis, combining the intermolecular and intramolecular information would provide clues toward clarifying the underlying sequence of events.

From an intermolecular point of view, anti-BCOADC-E1*α* antibodies were characterized as follows. First, they tended to appear in association with antibodies against other subunits constituting BCOADC but not PDC. As a result, a clustering of antibody reactivities toward BCOADC was observed that could be distinguished from that toward PDC. Second, the appearance of anti-BCOADC-E1*α* antibodies always accompanied that of anti-BCOADC-E2 antibodies, suggesting a constant order in their emergence. These observations are understandable by assuming that the native BCOADC complex is the immunogen with the E2 subunit as the primary determinant. Conceivably, the immune system may be erroneously induced to attack the self BCOADC complex released from damaged cells, consequently giving rise to intermolecular determinant spreading between the subunits within the complex. According to the literature, intermolecular determinant spreading is generally observed between the components of macromolecular complexes [31] and is characterized by the progressive and ordered appearance of autoreactivities against different components of the complexes [32, 33]. The above findings are compatible with this model, and thus anti-BCOADC autoimmunity is a plausible example of this amplification cascade. If this notion is true, breakdown of self-tolerance could be observed against the BCOADC-E1*α* subunit itself.

From an intramolecular point of view, the epitopes on BCOADC-E1*α* were characterized as follows. First, the major determinant recognized by all the sera with anti-BCOADC-E1*α* antibodies was composed of nested epitopes, indicating active responses against this region. Second, multiple epitopes were observed throughout the polypeptide, suggesting an antigen-driven mechanism. Third, the major epitope overlapped with the active center on the molecular surface, which seemed to be easily recognized by the surface immunoglobulins of B cells. Finally, antibody reactivities against human BCOADC-E1*α*-specific sequences were observed. Taken together, these observations indicate that the native BCOADC-E1*α* itself is targeted by the immune system. This feature extends the above-mentioned view of an antigen-driven mechanism, which may explain the causal relationship between the intermolecular and intramolecular determinant spreading.

There are many examples of autoimmune disorders in which the spreading of autoimmunity plays an active role in the disease pathology [14, 33]. Therefore, it is worth highlighting the underlying mechanisms whereby spreading takes place in anti-BCOADC-E1*α* autoimmunity. One of the possible causes of the diversification of the epitopes is molecular mimicry based on structural similarities between the self BCOADC-E1*α* antigen and an ortholog in either humans or pathogens [34]. However, the present study argues against the possibility of cross-reactivity between human BCOADC-E1*α* and human PDC-E1*α*, supporting independent autoimmunity against each E1*α* molecule. In addition, the anti-BCOADC-E1*α* antibodies were shown to recognize human-specific sequences. This is probably because the subunit composition and the amino acid sequence of BCOADC-E1 differ considerably among species. In particular, most gram-negative bacteria, including *Escherichia coli*, that have been proposed as causative agents for PBC [35, 36] are deficient in BCOADC [37]. Nevertheless, it may be possible that conformational epitopes are cross-reactive between human BCOADC-E1*α* and certain microbial antigens. In contrast to this lack of significant interspecies conservation of BCOADC-E1*α* at the primary structure level, the conservation is strong between the lipoyl-containing-E2 antigens, where molecular mimicry is considered highly likely [36].

Other than the molecular mimicry theory, the prevailing model emphasizes an immune regulatory role for B cells in determinant spreading [15, 38]. According to this model, B cell-mediated uptake of the multiprotein complex via surface immunoglobulins followed by processing and presentation to T cells is the underlying mechanism that leads to the breakdown of self-tolerance to each component of the complex. This concept depends on the physical associations among the antigen molecules and is fully compatible with our present observations. Thus, it can be hypothesized that this model explains the appearance of autoantibodies against the non-lipoyl-containing subunits, E1*α*, E1*β*, and E3, within the BCOADC and PDC complexes [9, 10, 39]. In contrast, however, the diversification of the autoimmunity between the lipoyl-containing E2 subunits of BCOADC and PDC could be well explained by the standard theory of molecular mimicry [36]. We speculate that the anti-E2 autoreactivity caused by mimicry may, in turn, act as an initiation factor that triggers the determinant spreading to other subunits within each complex. This scenario is consistent with the phenomenon termed “interparticle determinant spreading” between the macromolecular complexes in SLE [15, 40].

The current hypothesis on the etiopathogenesis of PBC implies that this disease is the result of a genetic predisposition that is permissive for still unknown environmental agents, similar to other autoimmune diseases [1, 41]. Recent genome-wide association studies have demonstrated that the HLA region is the most important genetic component for susceptibility to PBC, and that all of the other minor susceptibility variants are in immune-related genes, thereby further strengthening the similarity [42–44]. While determinant spreading is a well-known phenomenon in other autoimmune diseases, further investigations are required to clarify how the diversification of AMA is associated with the pathogenesis of PBC.

## Supplementary Material

Figure S1: The Z-score representation of reactivities of PBC sera against synthesized peptides of BCOADC-E1**α**. To normalize the results of pin ELISA (Figures 2a and 2b), the obtained OD450 values were transformed into Z-scores [25]. The pin number represents the N-terminal amino acid number of each peptide. The location of the major epitope is indicated by blue squares.
Figure S2: Solvent accessible surface areas of BCOADC-E1**α**. The solvent accessible surface areas of the BCOADC-1**α** subunit were calculated using the DSSP program (http://swift.cmbi.ru.nl/gv/dssp/) [26]. Two areas (aa 137-142 and 153-156) within the major determinant region are found to be accessible to solvents.
Figure S3: Inhibition of BCOADC activity by the anti-BCOADC-E1**α**-positive sera. The BCOADC activity was reconstituted *in vitro* by mixing purified E1, E2 and E3 components as described previously [24]. Before the assays, 1.25 **ε**g of E1 was incubated with the indicated sera at a 200-fold dilution for 3 min on ice. The activities were calculated as percentages of the control activity without serum. Sera from two patients (P07 and P13) strongly inhibit the BCOADC activity, while normal sera (N01 and N02) do not.
Figure S4: Three-dimensional mapping of the epitope regions on the BCOADC-E1**α** subunit monomer. The epitope regions determined by ELISA were mapped on the crystal structure of the BCOADC-E1**α** subunit monomer (PDB: 2bev) [21]. The residue numbers of each region are shown. The antibody reactivities are indicated in red and pink, which correspond to *+++* and *++* in Table 3, respectively.
Table S1: Numbers of patients with and without anti-PDC-E1**α** and anti-PDC-E2 antibodies. The appearance of anti-PDC-E1**α** antibodies was significantly linked to that of anti-PDC-E2 antibodies by Fisher's exact test (n = 30, P = 0.004).
Table S2: Core sequences of the epitopes defined by multipin ELISA. Synthesized peptides were tested for their reactivities with high-titer sera (P13 and P18). The amino acid sequences of the defined epitopes are represented by the single-letter code.
A list of the accession numbers for the BCOADC-E1**α** from various species. Amino acid sequences of these proteins were searched for homology with that of human BCOADC-E1**α** using the ClustalW2 program (http://www.ebi.ac.uk/Tools/clustalw2) [28].

## Figures and Tables

**Table 1:** Number of patients with and without anti-BCOADC-E1a and anti-BCOADC-E2 antibodies.

		Anti-BCOADC-E2		Total
		Positive	Negative	
Anti-BCOADC-E1a	Positive	16/16 (100.0%)	0/16 (0.0%)	16
	Negative	10/14 (71.4%)	4/14 (28.6%)	14

Total		26/30 (86.7%)	4/30 (13.3%)	30

Note: the incidences of the anti-BCOADC-E1a and anti-BCOADC-E2 antibodies are significantly linked to each other by Fisher's exact test (*P* = 0.037).

**Table 2:** Spearman correlation coefficients between the antibody titers against the individual subunits of BCOADC and PDC.

		BCOADC-E1*α*	BCOADC-E1*β*	BCOADC-E2	PDC-E1*α*	PDC-E2	PDC-E3BP
BCOADC-E1*α*	Correlation coefficient (*r*)	—	0.786^∗^	0.336^∗^	0.193	0.073	0.044
	*P* value		0.018	0.043	0.168	0.358	0.413
BCOADC-E1*β*	Correlation coefficient (*r*)	0.786^∗^	—	0.014	0.143	0.214	0.079
	*P* value	0.018		0.322	0.380	0.322	0.351
BCOADC-E2	Correlation coefficient (*r*)	0.336^∗^	0.014	—	0.193	0.144	0.303
	*P* value	0.043	0.322		0.167	0.237	0.630
PDC-E1*α*	Correlation coefficient (*r*)	0.193	0.143	0.193	—	0.339^∗^	0.655^∗∗^
	*P* value	0.168	0.380	0.167		0.042	0.000
PDC-E2	Correlation coefficient (*r*)	0.073	0.214	0.144	0.339^∗^	—	0.649^∗∗^
	*P* value	0.358	0.322	0.237	0.042		0.000
PDC-E3BP	Correlation coefficient (*r*)	0.044	0.079	0.303	0.655^∗∗^	0.649^∗∗^	—
	*P* value	0.413	0.351	0.630	0.000	0.000	

NOTE: *P < 0.05,  **P < 0.01.

**Table 3:** Clinical statuses of the PBC patients and their antibody binding profiles against each epitope region.

Patient No.	Asymptomatic/Symptomatic		Epitope no.^b^											
		Stage^a^	1	2	3	4	5	6	7	8	9	10	11	12
			(aa 3143)	(4969)	(7188)	(93104)	(107123)	(134168)	(171188)	(215231)	(233256)	(259266)	(280294)	(311327)
P07	Asymptomatic	1	+++	+	++			+++	+				+++	+
P13	Symptomatic	4	++	+	+	+	+	+++	+++	+	+	++	+	
P18	Symptomatic	2	++	+	+	+	++	+++	++	+++	++	+	+	
P20	Asymptomatic	ND					+	++	+	+	+			

NOTE: ^a^the stages were determined according to Scheuer's classification [30].

^b^PBC sera with anti-BCOADC-E1a antibodies were analyzed by multipin ELISA. The reactivities against each epitope are summarized.

Symbols represent *Z*-scores as follows: ++, >3.0; +, 2.0-3.0; , 02.0.

Abbreviations: aa, amino acid; ND, not determined.

**Table 4:** Number of patients with and without anti-BCOADC-E1a and anti-PDC-E1a antibodies.

		Anti-PDC-E1a		Total
		Positive	Negative	
Anti-BCOADC-E1a	Positive	7/16 (43.8%)	9/16 (56.3%)	16
	Negative	4/14 (28.6%)	10/14 (71.4%)	14

Total		11/30 (36.7%)	19/30 (63.3%)	30

Note: the incidences of the anti-BCOADC-E1a and anti-PDC-E1a antibodies are independent of each other by Fischers exact test (*P* = 0.466).
