# Supplementary material for: Characterization of Autoantibodies against the E1α Subunit of Branched-Chain 2-Oxoacid Dehydrogenase in Patients with Primary Biliary Cirrhosis
Source: Int J Hepatol. 2012 Jun 20;2012:369740. doi: 10.1155/2012/369740 (PMC3388300; doi:10.1155/2012/369740)
Supplement: Supplementary file 1 — Figure S1: The Z-score representation of reactivities of PBC sera against synthesized peptides of BCOADC-E1α. To normalize the results of pin ELISA (Figures 2a and 2b), the obtained OD450 values were transformed into Z-scores [25]. The pin number represents the N-terminal amino acid number of each peptide. The location of the major epitope is indicated by blue squares. Figure S2: Solvent accessible surface areas of BCOADC-E1α. The solvent accessible surface areas of the BCOADC-1α subunit were calculated using the DSSP program (http://swift.cmbi.ru.nl/gv/dssp/) [26]. Two areas (aa 137-142 and 153-156) within the major determinant region are found to be accessible to solvents. Figure S3: Inhibition of BCOADC activity by the anti-BCOADC-E1α-positive sera. The BCOADC activity was reconstituted in vitro by mixing purified E1, E2 and E3 components as described previously [24]. Before the assays, 1.25 εg of E1 was incubated with the indicated sera at a 200-fold dilution for 3 min on ice. The activities were calculated as percentages of the control activity without serum. Sera from two patients (P07 and P13) strongly inhibit the BCOADC activity, while normal sera (N01 and N02) do not. Figure S4: Three-dimensional mapping of the epitope regions on the BCOADC-E1α subunit monomer. The epitope regions determined by ELISA were mapped on the crystal structure of the BCOADC-E1α subunit monomer (PDB: 2bev) [21]. The residue numbers of each region are shown. The antibody reactivities are indicated in red and pink, which correspond to +++ and ++ in Table 3, respectively. Table S1: Numbers of patients with and without anti-PDC-E1α and anti-PDC-E2 antibodies. The appearance of anti-PDC-E1α antibodies was significantly linked to that of anti-PDC-E2 antibodies by Fisher's exact test (n = 30, P = 0.004). Table S2: Core sequences of the epitopes defined by multipin ELISA. Synthesized peptides were tested for their reactivities with high-titer sera (P13 and P18). The amino acid sequences of [file 369740.f1.pdf]

Supplementary Figures

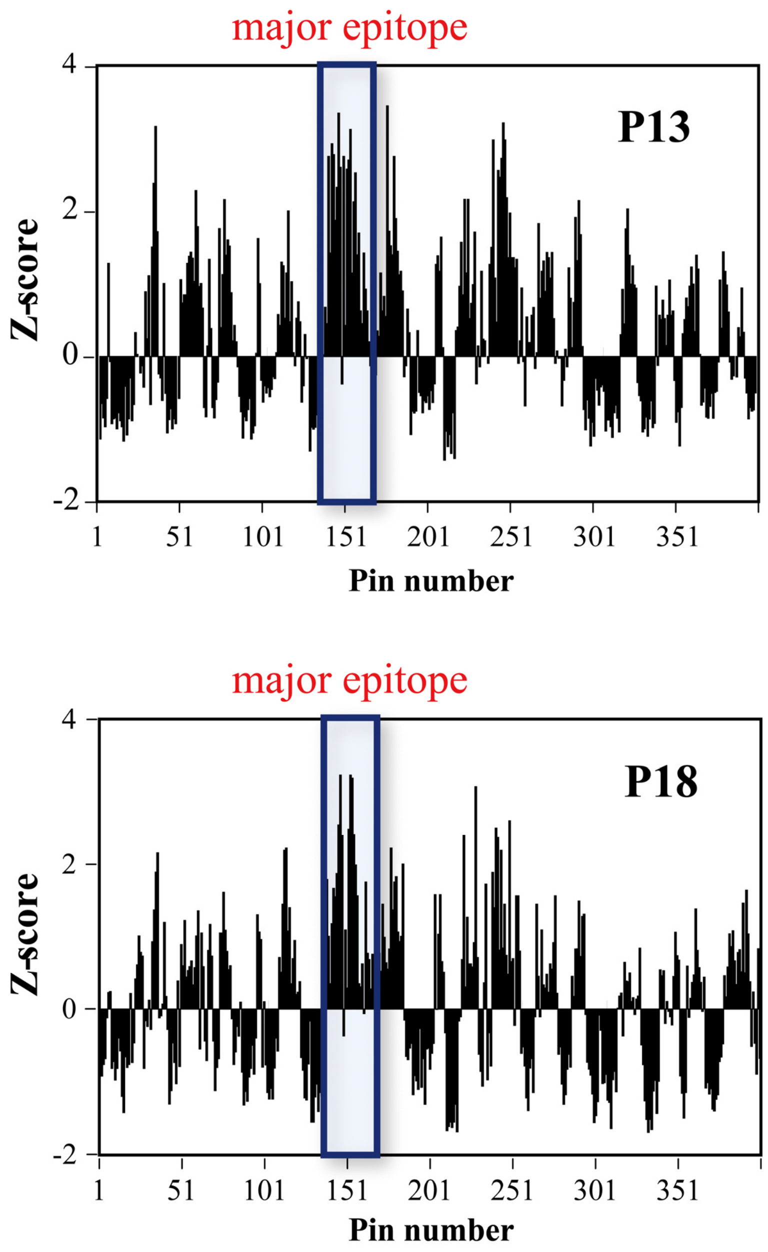

Figure S1. Z-score representation of the reactivities of PBC sera against synthesized peptides of BCOADC-E1α. To normalize the results of the pin ELISA (Figures 2a and 2b), the obtained OD450 values were transformed into Z-scores [25]. The pin number represents the N-terminal amino acid number of each peptide. The location of the major epitope is indicated by the blue squares.

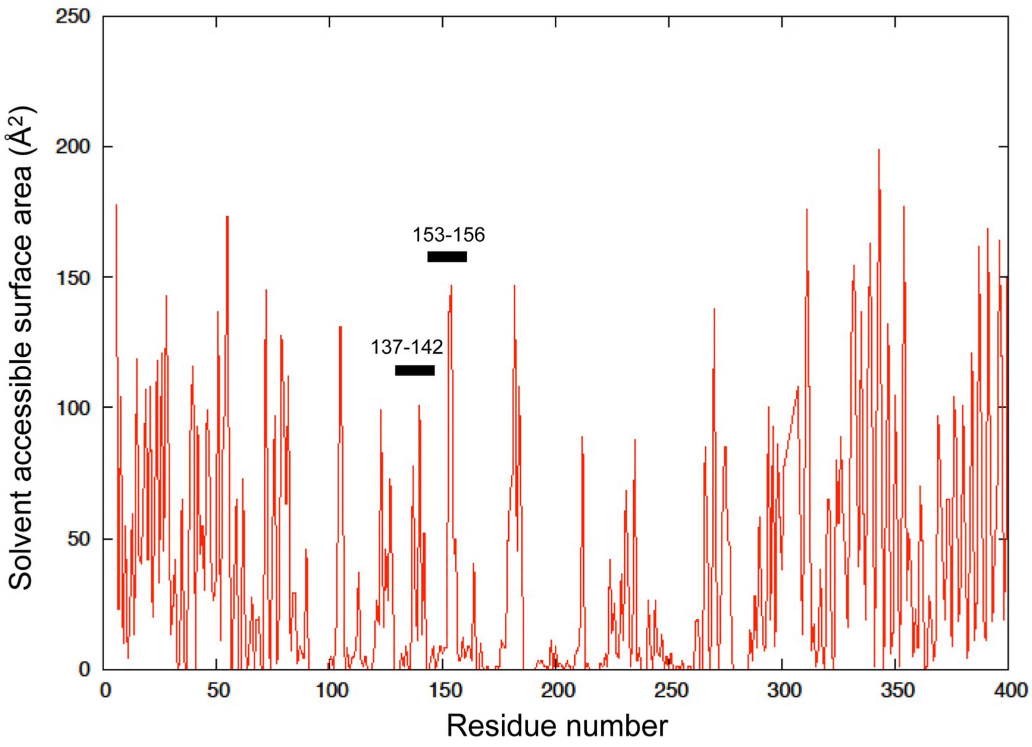

Figure S2. Solvent accessible surface areas of BCOADC-E1α. The solvent accessible surface areas of the BCOADC-1α subunit were calculated using the DSSP program (http://swift.cmbi.ru.nl/gv/dssp/) [26]. Two areas (aa 137-142 and 153-156) within the major determinant region are found to be accessible to solvents.

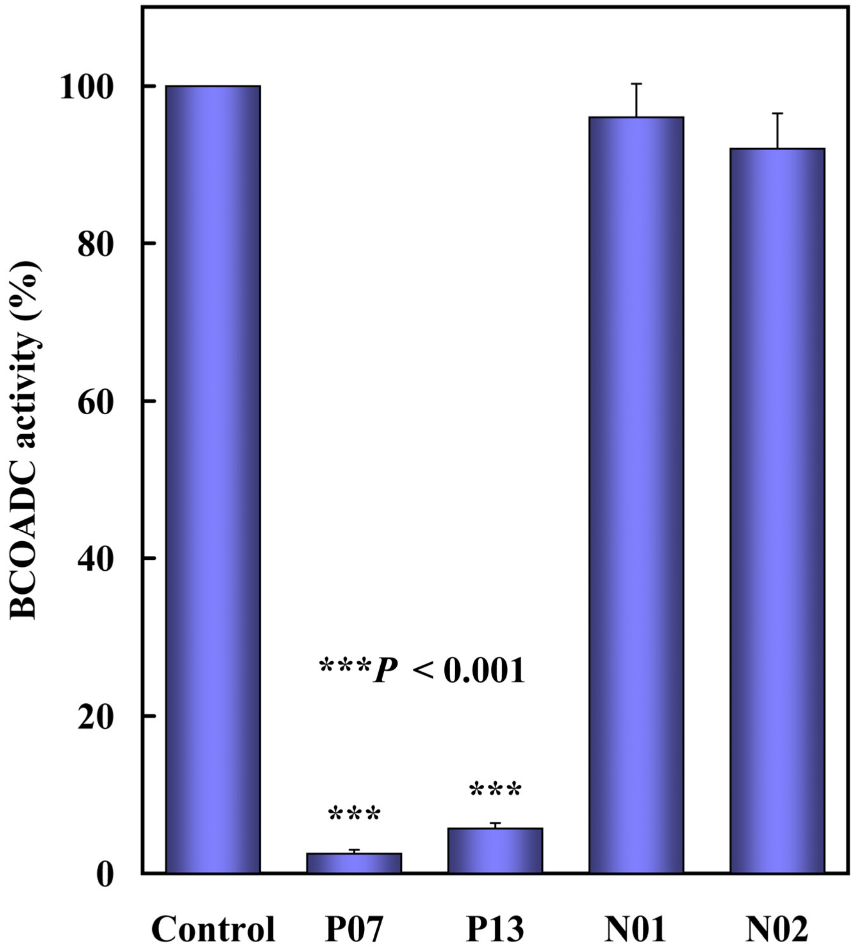

Figure S3. Inhibition of BCOADC activity by the anti-BCOADC-E1α-positive sera. The BCOADC activity was reconstituted *in vitro* by mixing purified E1, E2 and E3 components as described previously [24]. Before the assays, 1.25 μg of E1 was incubated with the indicated sera at a 200-fold dilution for 3 min on ice. The activities were calculated as percentages of the control activity without serum. Sera from two patients (P07 and P13) strongly inhibit the BCOADC activity, while normal sera (N01 and N02) do not.

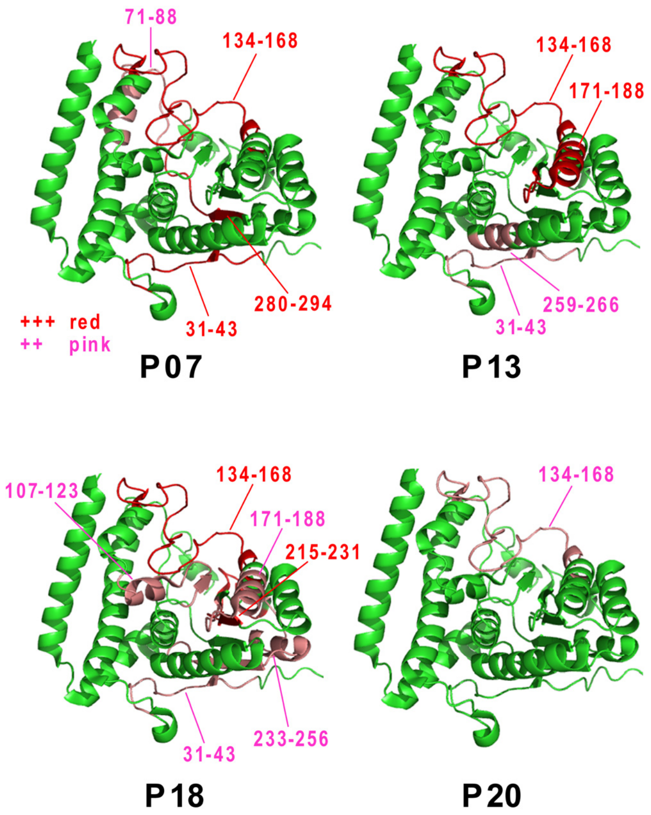

Figure S4. Three-dimensional mapping of the epitope regions on the BCOADC-E1α subunit monomer. The epitope regions determined by ELISA were mapped on the crystal structure of the BCOADC-E1α subunit monomer (PDB: 2bev) [21]. The residue numbers of each region are shown. The antibody reactivities are indicated in red and pink, which correspond to +++ and ++ in Table 3, respectively.

Supplementary Tables

Table S1. Numbers of Patients with and without Anti-PDC-E1α and Anti-PDC-E2 Antibodies

		Anti-PDC-E2		
		**Positive**	**Negative**	**Total**
Anti-PDC-E1α	Positive	11/11 (100.0%)	0/11 (0.0%)	11
	Negative	9/19 (47.4%)	10/19 (52.6%)	19
Total		20/30 (66.7%)	10/30 (33.3%)	30

NOTE: The incidences of the anti-PDC-E1α and anti-PDC-E2 antibodies are significantly linked to each other by Fisher's exact test (P = 0.004).

Table S2. Core Sequences of the Epitopes Defined by Multipin ELISA

Epitopes	Core sequences	Amino acids
1	YRVMDRQG	34-41
2	LKLYKSMT	58-65
3	YESQRQGR	75-82
4	THVGSAAA	95-102
5	YREAGVLM	113-121
6	ISDLGKGR<br>GRQMPVHY<br>YGCKERHF	137-144<br>143-151<br>150-157
7	VGAAYAAK<br>YAAKRANA	172-179<br>176-183
8	FCRNNGYA	218-225
9	RGDGIAAR<br>ARGPGYGI	235-242<br>241-248
10	FAVYNATK	259-266
11	MTYRIGHH	284-291
12	PISRLRHY	315-322

NOTE: Synthesized peptides were tested for their reactivities with high-titer sera (P13 and P18) as described in the text. The amino acid sequences of the defined epitopes are represented by the single-letter code.

Supplementary Methods

Supplementary information for the homology analysis

The accession numbers for the proteins used in the homology analysis were as follows: *Homo sapiens*, NP_000700; *Mus musculus*, NP_031559; *Gallus gallus*, BAB32665; *Leishmania major*, CAC03559; *Enterococcus faecalis*, AAD55377; *Streptomyces avermitilis*, AAB03377; *Rattus norvegicus*, AAA40811; *Bacillus subtilis*, AAA22278; *Bos taurus*, AAA30595; *Drosophila melanogaster*, AAF54398; *Caenorhabditis elegans*, NP_499693; *Brucella melitensis*, AAL53990; *Pseudomonas aeruginosa*, AAG05635; *Pseudomonas putida*, DEPSXA; *Mycobacterium tuberculosis*, AAK46876; *Novosphingobium aromaticivorans*, ABD25490.
